# The green tea catechin epigallocatechin gallate induces cell cycle arrest and shows potential synergism with cisplatin in biliary tract cancer cells

**DOI:** 10.1186/s12906-015-0721-5

**Published:** 2015-06-23

**Authors:** Christian Mayr, Andrej Wagner, Daniel Neureiter, Martin Pichler, Martin Jakab, Romana Illig, Frieder Berr, Tobias Kiesslich

**Affiliations:** Department of Internal Medicine I, Salzburger Landeskliniken – SALK, Paracelsus Medical University, Salzburg, Austria; Laboratory for Tumour Biology and Experimental Therapies, Institute of Physiology and Pathophysiology, Paracelsus Medical University, Salzburg, Austria; Institute of Pathology, Salzburger Landeskliniken – SALK, Paracelsus Medical University, Salzburg, Austria; Department of Experimental Therapeutics, The University of Texas MD Anderson Cancer Center, Houston, Texas USA; Laboratory for Functional and Molecular Membrane Physiology, Institute of Physiology and Pathophysiology, Paracelsus Medical University, Salzburg, Austria

**Keywords:** EGCG, Green tea, Biliary tract cancer, Drug synergism, Cisplatin, Cell cycle arrest

## Abstract

**Background:**

The green tea catechin epigallocatechin gallate (EGCG) was shown to effectively inhibit tumor growth in various types of cancer including biliary tract cancer (BTC). For most BTC patients only palliative therapy is possible, leading to a median survival of about one year. Chemoresistance is a major problem that contributes to the high mortality rates of BTC. The aim of this study was to investigate the cytotoxic effect of EGCG alone or in combination with cisplatin on eight BTC cell lines and to investigate the cellular anti-cancer mechanisms of EGCG.

**Methods:**

The effect of EGCG treatment alone or in combination with the standard chemotherapeutic cisplatin on cell viability was analyzed in eight BTC cell lines. Additionally, we analyzed the effects of EGCG on caspase activity, cell cycle distribution and gene expression in the BTC cell line TFK-1.

**Results:**

EGCG significantly reduced cell viability in all eight BTC cell lines (p < 0.05 or p < 0.01, respectively, for most cell lines and EGCG concentrations > 5 μM). Combined EGCG and cisplatin treatment showed a synergistic cytotoxic effect in five cell lines and an antagonistic effect in two cell lines. Furthermore, EGCG reduced the mRNA levels of various cell cycle-related genes, while increasing the expression of the cell cycle inhibitor p21 and the apoptosis-related death receptor 5 (p < 0.05). This observation was accompanied by an increase in caspase activity and cells in the sub-G1 phase of the cell cycle, indicating induction of apoptosis. EGCG also induced a down-regulation of expression of stem cell-related genes and genes that are associated with an aggressive clinical character of the tumor, such as cd133 and abcg2.

**Conclusions:**

EGCG shows various anti-cancer effects in BTC cell lines and might therefore be a potential anticancer drug for future studies in BTC. Additionally, EGCG displays a synergistic cytotoxic effect with cisplatin in most tested BTC cell lines.

**Graphical abstract:**
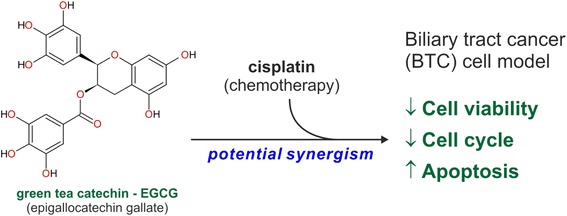
Summary illustration

## Background

Biliary tract cancer (BTC) develops at different locations within the biliary tree system. The prognosis for BTC remains very poor with a five year survival rate of only 10 % [1]. Due to the silent clinical character of the malignancy, BTC patients are often diagnosed at late stages and only few patients are candidates for surgical resection – the currently only curative treatment option. Palliative chemotherapeutic therapy with cisplatin and gemcitabine achieves an average survival of about one year [1, 2]. In general, high recurrence rates, the lack of suitable follow-up therapies and fast development of chemoresistance impede significant therapeutic success [3]. Therefore, new therapeutic approaches that inhibit BTC tumorigenesis and that may also overcome or even reverse the chemoresistance of BTC cells are needed.

Green tea is a very popular beverage that had its origin in China several thousand years ago. Numerous studies prove that green tea consumption has cancer-preventive effects (reviewed in [4]). Green tea contains several catechins of which epigallocatechin gallate (EGCG) is the most abundant that furthermore shows the strongest anti-cancer effects [5, 6]. Previous studies demonstrated that EGCG inhibited growth of cancer cells by causing apoptosis and cell cycle arrest in various types of cancer, such as lung cancer [7], prostate cancer [8–10], head and neck squamous carcinoma [11], hepatocellular carcinoma [12] as well as breast, colon, skin and bladder cancer [4] *in vitro* and *in vivo*. There is also evidence that EGCG has anti-cancer effects in BTC: two studies demonstrated that EGCG suppresses the growth and invasion potential of various human BTC cell lines [13, 14]. Lang et al. showed that treatment with EGCG sensitized BTC cells to apoptosis caused by chemotherapeutics gemcitabine, mitomycin C or 5-fluorouracil. Additionally, EGCG reduced BTC tumor growth and increased sensitivity to gemcitabine *in vivo*. They also demonstrated, that combined EGCG and gemcitabine incubation was associated with a synergistic cytotoxic effect [15]. This potential synergistic effect of EGCG with other chemotherapeutic compounds was also seen for the combination with vorinostat, a histone deacatylase inhibitor, in human HuCC-T1 cholangiocarcinoma cells [16]. These studies make EGCG not only an interesting substance for single, but also a potentially attractive adjuvant substance for combined therapy of BTC *in vitro* and *in vivo* [5].

Based on these encouraging preliminary results and a paucity of data about a potential synergism of EGCG and cisplatin in BTC cells, we hypothesized that combined treatment of EGCG with cisplatin shows a synergistic cytotoxic effect. For this purpose, we used a comprehensive approach by testing combined EGCG and cisplatin treatment in a panel of eight different BTC cell lines. Since previous studies suggest that EGCG exhibits diverse anti-cancer effects, we explored the EGCG-caused changes in cell-cycle distribution, caspase activity and gene expression of selected cell cycle- and apoptosis-related genes as well as genes that are associated with an aggressive tumor character and potential cancer stem cell (CSC) status.

## Methods

### Substances and cell culture

EGCG was obtained from Sigma Aldrich (Vienna, Austria) and dissolved in H_2_O to a stock concentration of 10 mM and stored in aliquots at -20 °C. Cisplatin was provided by the hospital’s pharmacy (Landesapotheke, Salzburger Landeskliniken) as a stock solution of 3.33 mM and was stored at 4 °C. Resazurin was purchased from Sigma Aldrich and dissolved in Dulbecco’s Phosphate Buffered Saline (DPBS, Sigma Aldrich). Overall five bile duct carcinoma cell lines CCSW-1 (G2 [17]), BDC (G4 [18]), EGI-1 (G3, [19]), SkChA-1 (G3, [20]), TFK-1 (G2, [21]) and three gallbladder cancer cell lines MzChA-1 (G1 [20]), MzChA-2 (G2 [20]) and GBC (G1 [22]) were cultured in high glucose Dulbecco’s modified Eagle’s medium (DMEM; Gibco, Life Technologies) supplemented with 10 % (v/v) foetal bovine serum (FBS; Gibco, Life Technologies) as described before [23, 24] and are together termed as BTC cell lines [25]. For seeding we used the following cell numbers per cm^2^ of the culture receptacle in 10 % FBS DMEM: 3.95*10^4^ (BDC, MzChA-2), 4.74*10^4^ (CCSW-1, GBC), 5.53*10^4^ (SkChA-1), 6.32*10^4^ (EGI-1, TFK-1), and 7.11*10^4^ (MzChA-1). For EGCG, cisplatin and combined drug treatment we used serum-free DMEM (sfDMEM) to avoid possible interactions of the drugs with components of the serum.

### Drug cytotoxicity

We investigated the cell line- and dose-dependent cytotoxic effect of EGCG only and combined EGCG cisplatin treatment on cells grown in 96-well microplates. Quantification of cell viability was carried out using the resazurin assay and an Infinite M200 microplate reader (Tecan, Groedig, Austria) as described [24, 26]. Cells were treated with a dilution series of EGCG (0.2-400 μM) in sfDMEM for 72 h based on previously published concentration ranges [14–16]. Viability was related to untreated cells (sfDMEM only) samples. For combined EGCG and cisplatin treatment, cells were incubated in sfDMEM for 72 h with various concentrations of each drug alone (EGCG: 5, 20, 50 and 80 μM; cisplatin: 10, 20, 40 and 80 μM; data only shown for 20 μM EGCG, 50 μM EGCG and 40 μM cisplatin, respectively) and two combinations (20 μM EGCG + 40 μM cisplatin; 50 μM EGCG + 40 μM cisplatin). For drug combination experiments, cells were simultaneously incubated with sfDMEM containing either single or combined drugs. Viability was measured using the resazurin assay and an Infinite M200 microplate reader (Tecan) and viability was related to untreated cells (sfDMEM only) samples. To evaluate potential synergistic cytotoxic effects of combined EGCG and cisplatin treatment, we calculated the combination index (CI) using the CompuSyn software (www.combosyn.com). As described by Chou [27], combinations that lead to a CI greater than 1.1 are termed as antagonistic, combinations that lead to a CI less than 0.9 are termed as synergistic and combinations that lead to a CI between 0.9 and 1.1 are termed as additive.

### Gene expression analysis

TFK-1 cells were treated with 50 μM EGCG in sfDMEM in 60 mm cell culture dishes for 24 h. Total RNA was isolated using the RNeasy Kit and QIAshredder tubes (Qiagen). cDNA synthesis was done with the ImProm-II™ Reverse Transcription System (Promega). Quantification of gene expression was performed by quantitative real time reverse transcription PCR (qRT-PCR) using the GoTaq qPCR Master Mix (Promega) and a ViiA7 real time PCR system (Applied Biosystems, Life Technologies). All samples were measured at least in biological triplicates. Melting curve analysis was performed for each sample and primer pair to verify specificity of PCR products. Samples were normalized to beta-actin and treated samples were normalized to untreated controls according to the ΔΔCt method [28]. Down-regulated genes (ΔΔCt < 1) are represented as –(1/fold change) for a more clear visualization.

### Caspase

TFK-1 cells were seeded in 96-well microplates and incubated with 50 μM EGCG in sfDMEM for 24, 48 and 72 h, respectively. Apoptosis analysis was performed using the Caspase-Glo® 3/7 Assay (Promega) according to the manufacturer’s protocol with an Infinite M200 microplate reader (Tecan). For each time point, caspase activity was related to a corresponding untreated (sfDMEM only) control. Microscopic pictures were taken with an inverted phase contrast microscope (Motic AE31) which was equipped with a CCD-1300B digital camera (Allied Vision Technologies/VDS Vosskühler, Stadtroda, Germany) controlled by LUCIA imaging software (Laboratory Imaging Systems, Prague, Czech Republic).

### Cell cycle analysis

For cell cycle analysis, TFK-1 cells were seeded in 30 mm cell culture dishes for 72 h in sfDMEM medium containing 50 μM EGCG. After 72 h cells were harvested with 1x Trypsin-EDTA (0.5 %; Gibco, Life Technologies) and resuspended in 100 μL DBPS. Cells were fixed by addition of 1 ml 75 % ice-cold EtOH and incubated for 15 min on ice. After centrifugation, cells were resuspended in 200 μL DBPS containing 0.04 mg/ml propidium iodide (Sigma Aldrich, Fluka) and 0.1 mg/ml RNase (Ribonuclease A from bovine pancreas, Sigma Aldrich) and incubated for 30 min at 37 °C protected from light. After addition of 500 μl DPBS, cell cycle analysis was carried out on a Quanta SC flow cytometer (Beckman Coulter, Krefeld, Germany). For data analysis FlowJo software was used (Ashland, Oregon, USA) [29].

### Statistics

All data points represent mean values of at least three biological replicates ± SEM. Paired student’s *t*-test was used for calculation of significance between groups. All calculations were performed using OriginPro 9.1 (OriginLab, Northampton, MA, USA). Statistical results were considered significant (*) or highly significant (**) at p < 0.05 and p < 0.01, respectively.

## Results

### EGCG reduces cell viability in eight BTC cell lines

**Fig. 1:**
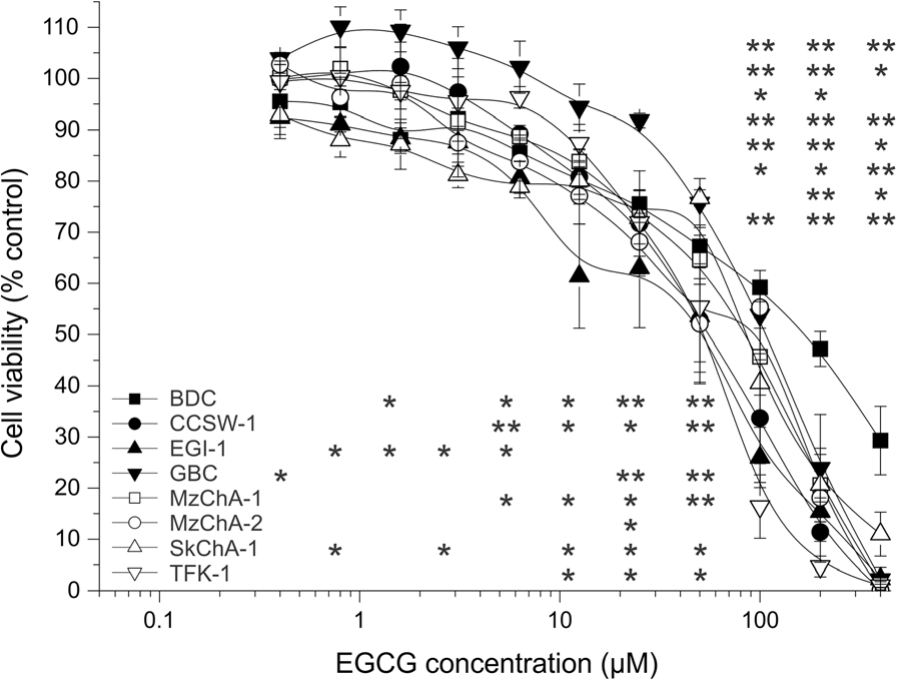
Cell line- and dose-dependent effect of EGCG on cell viability of BTC cell lines. Dose-dependent effect of 72 h EGCG treatment on eight BTC cell lines related to untreated control. Asterisks indicate significant (*, p < 0.05) or highly significant (**, p < 0.01) differences in viability related to untreated control cells. Abbreviations: BTC: Biliary tract cancer, EGCG: Epigallocatechin gallate, h: hours

Treatment of eight BTC cell lines with different concentrations of EGCG (range: 0.2-400 μM) for 72 h significantly reduced the cell viability in all cell lines in a dose- and cell line-dependent manner (Fig. 1). Interestingly, for all cell lines, except for GBC, cell viability noticeably declined for EGCG concentrations between 12.5 and 25 μM (for GBC between 50 and 100 μM). Treatment with the highest concentration of EGCG (400 μM) reduced the cell viability to almost 0 % for six cell lines (CCSW-1, EGI-1, GBC, MzChA-1, MzChA-2 and TFK-1) whereas for BDC and SkChA-1, a certain percentage of cells (approximately 30 % for BDC and 10 % for SkChA-1, respectively) survived (Fig. 1).

### Synergistic effect of EGCG and cisplatin treatment

**Fig. 2:**
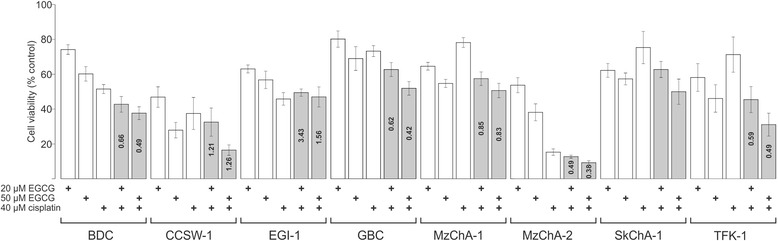
Combined EGCG and cisplatin treatment in BTC cell lines. Potential synergistic cytotoxic effects of combined EGCG and cisplatin treatment were evaluated in eight BTC cell lines. Cell viability is represented relative to untreated control. For SkChA-1 cells, values from single treatments were suitable for CI calculation. Single treatments, as well as drug combinations are indicated as “+”. Based on [27], the CI was calculated: a CI less than 0.9 represents a synergistic, a CI greater than 1.1 an antagonistic and a CI between 0.9 and 1.1 an additive effect. CI values are shown within the bars of the drug combinations. Abbreviations: BTC: Biliary tract cancer, CI: combination index, EGCG: Epigallocatechin gallate

To assess a potential synergistic effect, we measured the cell viability for single and combined treatments and calculated the combination index (CI). Combined EGCG and cisplatin treatment caused an antagonistic effect in two cell lines (CCSW-1 and EGI-1) for both tested combinations. For one cell line (SkChA-1) the data points achieved by single treatments were not suitable for CI calculation. The remaining five BTC cell lines showed a clear synergistic effect (BDC, GBC, MzChA-2, TFK-1; CI range 0.38 to 0.66) or a moderate synergistic effect (MzChA-1; CI range 0.83 to 0.85), respectively, for combined EGCG and cisplatin treatment (Fig. 2). Interestingly, the categorization (synergistic versus antagonistic)of the CI remained the same for both combinations (20 μM EGCG and 40 μM Cis; 50 μM EGCG and 40 μM Cis) for all cell lines with a synergistic and antagonistic CI, respectively.

### Caspase activity

**Fig. 3:**
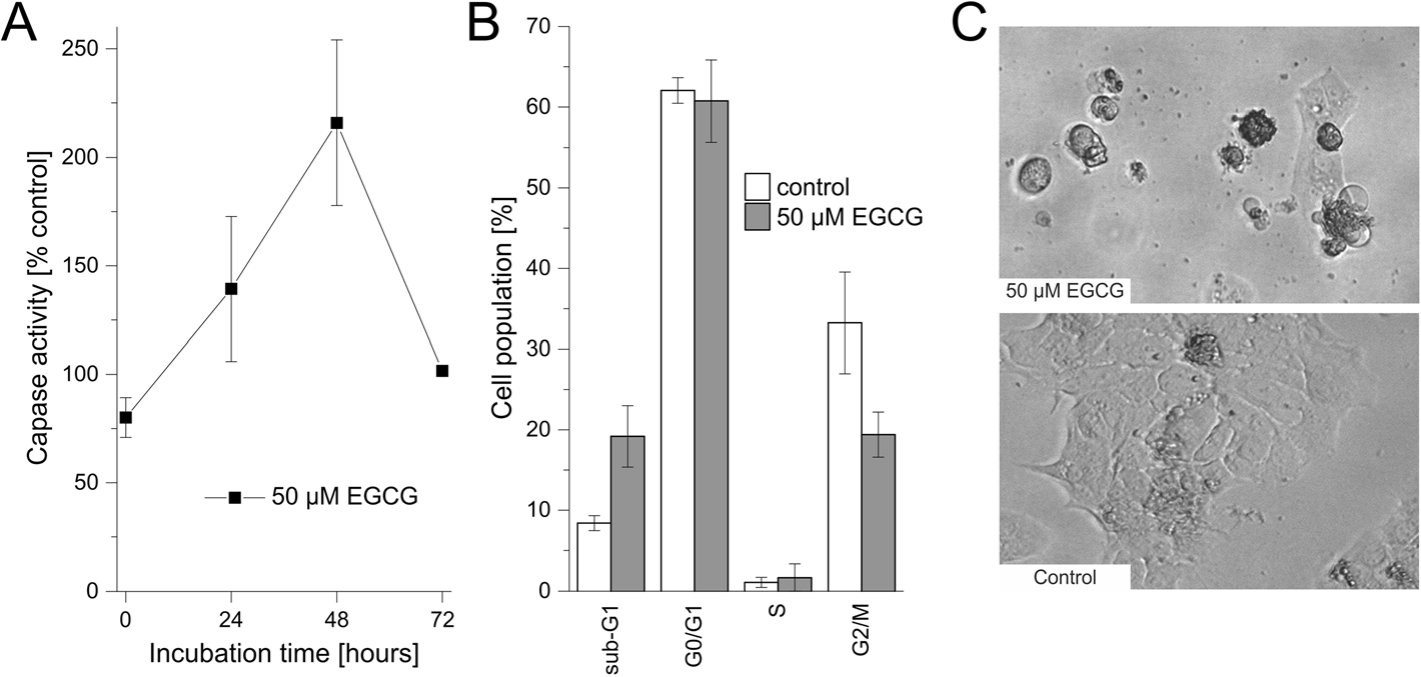
Effect of EGCG on caspase activity and cell cycle in TFK-1 cells. **a** Time-dependent effect of 50 μM EGCG on caspase activity in TFK-1 cells. For each time point, values are related to untreated control. **b** Cell cycle distribution of TFK-1 cells after 72 h. Sub-G1 represents cells with a DNA content less than 2 N, G0/G1 cells with DNA content 2 N, S cells with DNA content greater than 2 N and G2/M cells with DNA content 4 N. **c** Representative microscopic pictures of EGCG-treated and untreated TFK-1 cells, showing cell shrinkage, rounding and apoptotic bodies, respectively. Abbreviations: EGCG: Epigallocatechin gallate

Previous studies showed that EGCG causes apoptosis in cancer cells [30]. Treatment of TFK-1 cells with 50 μM EGCG caused a steady increase of caspase activity after 24 h and 48 h, respectively, whereas after 72 h caspase activity was reduced to its original value (Fig. 3a). This suggests that EGCG is able to slightly induce apoptosis in TFK-1 cells.

### Cell cycle distribution

To confirm the increase in apoptosis we performed cell-cycle analysis of TFK-1 cells. EGCG treatment increased the percentage of cells in sub-G1 (from 8.4 % to 19.2 %), again indicating apoptosis. Additionally, we observed a decrease of cells in G2/M (from 33.3 % to 19.4 %). The overall percentage of cells in G0/G1 and S-phases remained unchanged (Fig. 3b).

### Gene expression

**Fig. 4:**
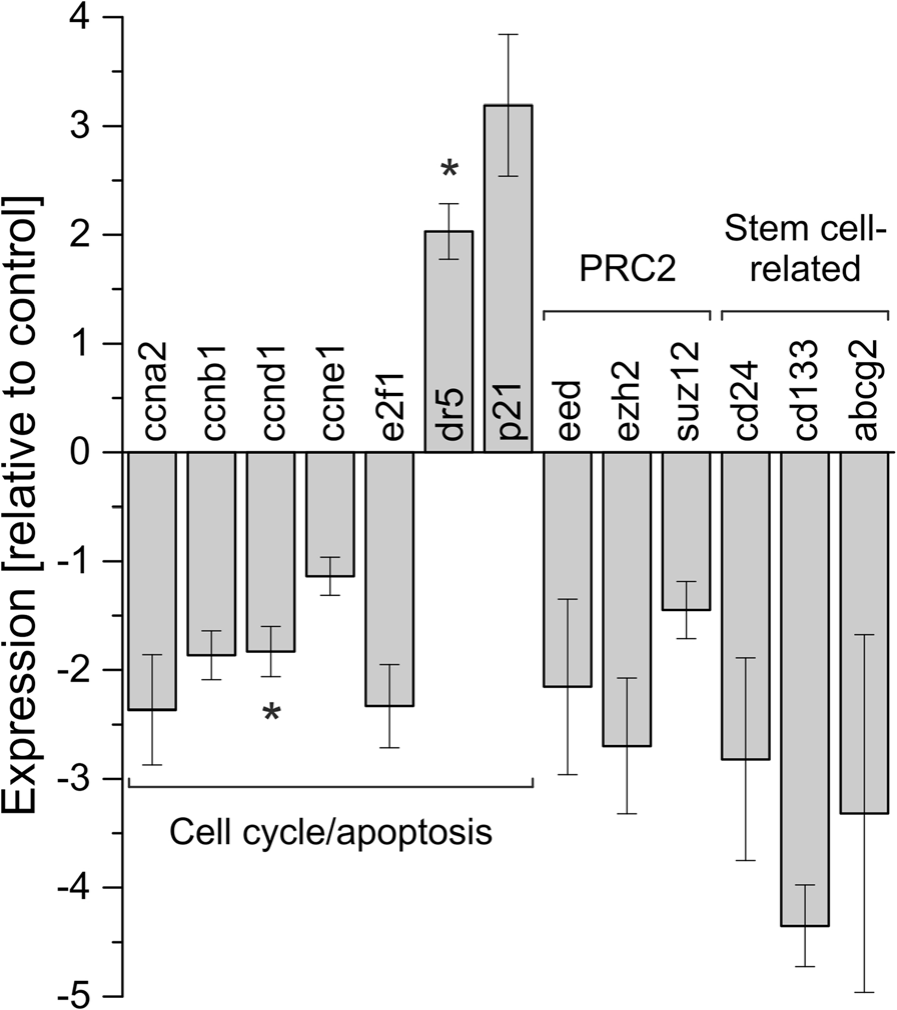
Effect of EGCG on gene expression in TFK-1 cells. TFK-1 cells were treated with 50 μM EGCG for 24 h. All samples were normalized to beta-actin and mRNA levels are presented relative to untreated controls according to the ΔΔCt method [28]. Asterisks indicate significant (p < 0.05) differences. Abbreviations: abcg2: ATP-binding cassette, sub-family G, member 2; ccna2: cyclin A2; ccnb1: cyclin B1; ccnd1: cyclin D1; ccne1: cyclin E1; cd133: prominin 1; cd24: CD24 molecule; dr5: tumor necrosis factor receptor superfamily, member 10b; e2f1: E2F transcription factor 1; eed: embryonic ectoderm development; EGCG: Epigallocatechin gallate; ezh2: enhancer of zeste homolog 2; h: hours, p21: Cyclin-Dependent Kinase Inhibitor 1A; suz12: SUZ12 polycomb repressive complex 2 subunit

In a last step we investigated the effect of EGCG treatment on the expression of cell cycle- (ccna2, ccnb1, ccnd1, ccne1, e2f1) and apoptosis-related genes (dr5, p21), genes that play a role in a potential CSC phenotype (cd24, cd133) and multidrug resistance (abcg2), as well as genes that are related to general enhanced aggressiveness of BTC (eed, ezh2, suz12): EGCG reduced the expression of ccna2, ccnb1, ccnd1 and e2f1, but not of ccne1 in TFK-1 cells. This was accompanied by an enhanced level of dr5 and p21 gene expressions. In addition, we saw a reduction of mRNA levels of cd24 and cd133 as well as of abcg2 and e2f. Interestingly, we also observed a decline of the expression of the three core components of the polycomb repressive complex 2 (PRC2, a major epigenetic regulator) eed, ezh2 and suz12 to different extent (Fig. 4).

## Discussion

In our study we investigated the effect of EGCG treatment alone and in combination with the standard chemotherapeutic cisplatin on the viability of eight different BTC cell lines. Furthermore we studied the effects of EGCG on caspase activity, cell cycle and gene expression of genes related to different aspects of BTC tumorigenesis in the established BTC cell line TFK-1 [21].

Incubation with EGCG led to a significant reduction of cell viability in all eight BTC cell lines. The effect was concentration-dependent in most cell lines. At the highest concentration of 400 μM, for six cell lines (CCSW-1, EGI-1, GBC, MzChA-1, MzChA-2 and TFK-1) almost no vital cells were measurable, whereas for BDC and SkChA-1 a certain amount of cells survived, indicating different responsiveness to EGCG treatment. These findings are in line with a study carried out by Takada and colleagues, which showed that EGCG inhibited cell growth in a dose-dependent manner in three BTC cell lines (TGBC-2, SkChA-1 and NOZC-1) [13]. Furthermore, Lang et al. presented that EGCG not only reduced *in vivo* growth of MzChA-1 cells in a mouse model, but also that EGCG had no cytotoxic effect on non-malignant human cholangiocytes [15].

It is also known from previous studies that EGCG exhibits synergistic effects with other anticancer compounds [8, 31–34]. As for BTC, studies showed that EGCG was able to increase toxicity of the histone deacetylase inhibitor vorinostat [16] and the chemotherapeutics gemcitabine, mitomycin and 5-fluorouracil [15], respectively. Since, to our knowledge, no published data describe the combination of EGCG and the standard chemotherapeutic cisplatin in BTC cells, we asked the question if this combination acts synergistically. To distinguish additive from real synergistic cytotoxic effects of combined EGCG and cisplatin treatment we used the CI based on the study by Chou [27]. For five cell lines (BDC, GBC, MzChA-1, MzChA-2 and TFK-1) we saw a synergistic effect, whereas for two cell lines (CCSW-1 and EGI-1) we obtained an antagonistic effect of combined EGCG and cisplatin treatment (for SkChA-1 the measured values for single treatments were not suitable for CI calculation). These data indicate that the cytotoxic effect of EGCG and cisplatin co-treatment is highly cell-line dependent and can lead to either synergistic or antagonistic effects. Similar results were also observed in non-small cell lung cancer cell lines where the efficacy of cisplatin was enhanced by EGCG in one cell line, but antagonized in another cell line [31]. One suggested mechanism that may lead to a synergistic effect of EGCG and cisplatin co-treatment is that EGCG sensitizes (cisplatin-resistant) cancer cells to cisplatin treatment [7, 32]. Chemoresistant cells often over-express multidrug-resistance genes, such as abcg2, an ATP-binding cassette subfamily G2 member, that also might play a role in CSC [35]. Lee et al. showed that in head and neck squamous carcinoma, EGCG enhanced cisplatin efficacy by down-regulation of abcg2 [11]. In line with these results, EGCG reduced abcg2 mRNA expression in TFK-1 cells in the present study, which may explain the observed synergistic cytotoxic effect of combined EGCG and cisplatin treatment.

The cytotoxic effect of EGCG has in part been ascribed to cell cycle arrest [4, 9]. In our study, EGCG caused down-regulation of cell cycle-promoting genes ccna2, ccnb1, ccnd1 and e2f1, whereas the expression of the cell cycle inhibitor p21 and the apoptosis-related gene dr5 were up-regulated. These effects on mRNA expression were accompanied by a moderate increase of caspase activity, as well as an increase in sub-G1 population (indicating apoptosis) and a decrease in G2/M (indicating cell cycle arrest), respectively. One potential underlying mechanism might be the down-regulation of ccnd1 in the early phases of the cell cycle, inhibiting e2f1 to attain its active form, which in turn prevents the activation of ccna2, leading to cell cycle arrest and apoptosis. Another hypothesis could base on up-regulation of p21 which also inhibits ccna2 activation, again leading to cell cycle arrest and apoptosis [36]. Interestingly, we did not recognize an increase of p53 mRNA levels (data not shown), which suggests that the potential p21-mediated effect on the cell cycle may be independent of p53 or that the effect is executed by p21 rather than p53 as already shown in another study [9].

EGCG also slightly reduced mRNA levels of the two stem cell-related genes cd24 and cd133, known to be associated with enhanced aggressiveness, higher tumorigenic potential and stem cell status in BTC [37–39]. We also recognized that EGCG down-regulated the three core components of the PRC2 complex (eed, ezh2 and suz12), which is a major epigenetic regulator that performs trimethylation of histone 3 at lysine 27. Ezh2 is the enzymatically active molecule of this complex and was found to be over-expressed in BTC specimen and connected to poor clinical features as recently reviewed [40]. Interestingly, EGCG treatment had the strongest effect on ezh2 expression in the present study. This goes in line with the results presented by Choudhury and colleagues, who showed that EGCG alone and in combination with the PRC2 inhibitor 3-Deazaneplanocin A effectively reduced PRC2 activity in skin cancer cells [41].

## Conclusions

EGCG is a potent drug for treatment of BTC *in vitro*. EGCG significantly reduced cell viability of all eight BTC cell lines and for most cell lines a synergistic cytotoxic effect could be shown, when combined with the standard chemotherapeutic drug cisplatin. Additionally, EGCG showed a profound effect on the cell cycle, which changes were confirmed indirectly by down-regulation of various cell cycle-dependent genes, enhanced caspase activity and cell cycle arrest. Furthermore, EGCG might also have an effect on cancer stem cell properties. All these results suggest that EGCG may be a promising and versatile (adjuvant) drug for future *in vivo* experiments concerning BTC. Several completed or currently active and recruiting clinical phase I or II trials (n > 20) include EGCG as a chemopreventive or supportive drug in various tumor entities (www.clinicaltrials.gov). Therefore, future clinical studies need to fully evaluate the potential of the green tea constituent EGCG as an adjuvant or chemopreventive drug for BTC.
